# Supplementary figures and images for: Optimizing target-to-total DNA ratio in eDNA studies: effects of sampling, preservation, and extraction methods on single-species detection
Source: PeerJ. 2025 Oct 30;13:e20127. doi: 10.7717/peerj.20127 (PMC12579850; doi:10.7717/peerj.20127)

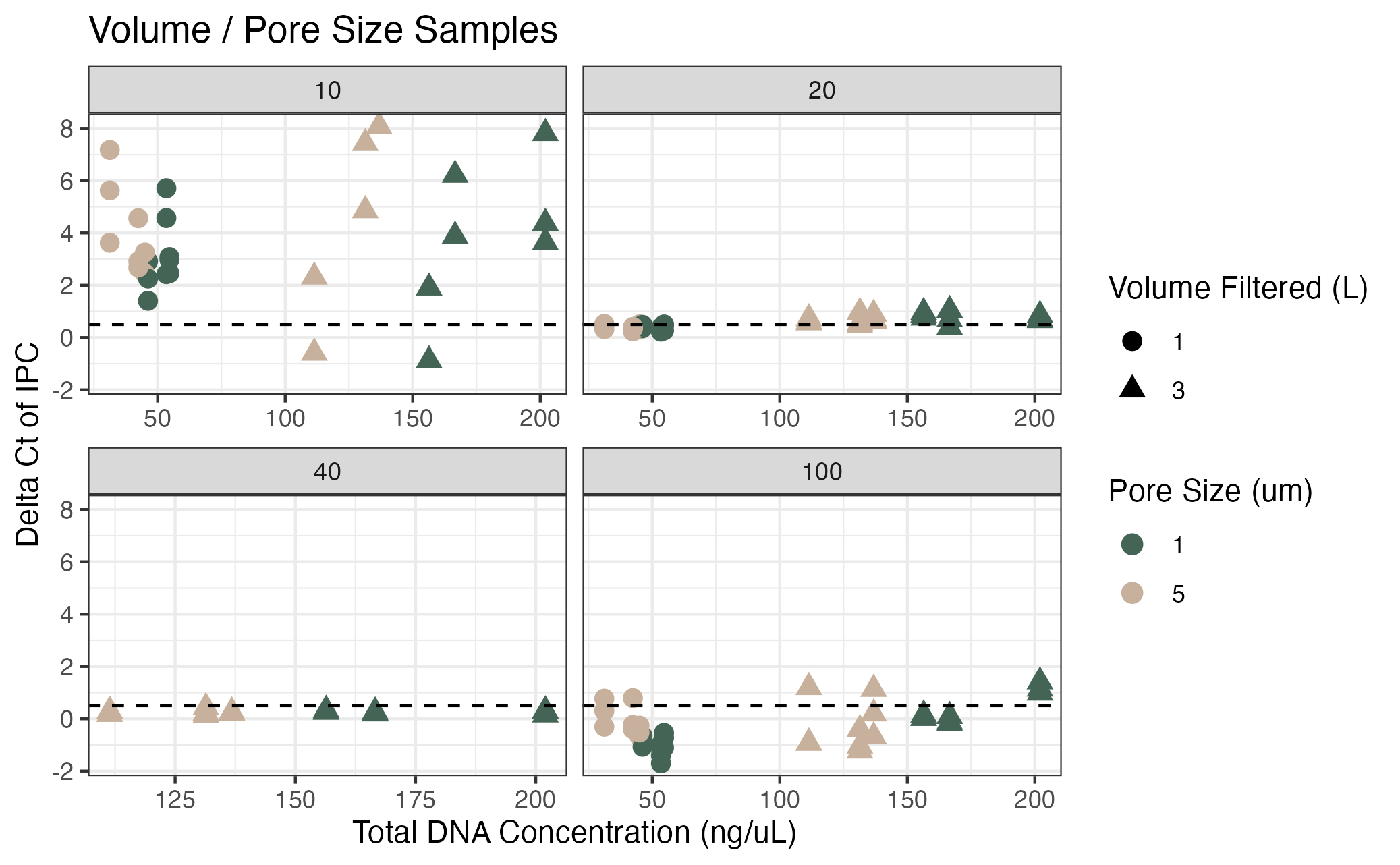

Supplement: Supplemental Information 1 — The y axis shows the difference in Ct value from the environmental sample versus the no template control of the spiked internal positive control (IPC). The x axis shows the total DNA concentration of the sample as measured by Qubit. Colors correspond to the pore size of the filter (um) and shapes correspond to the volume of water filtered (L). Dashed lines represent the threshold at which samples were deemed inhibited (0.5 Ct difference). Facets indicate the dilution factor. [file peerj-13-20127-s001.png]

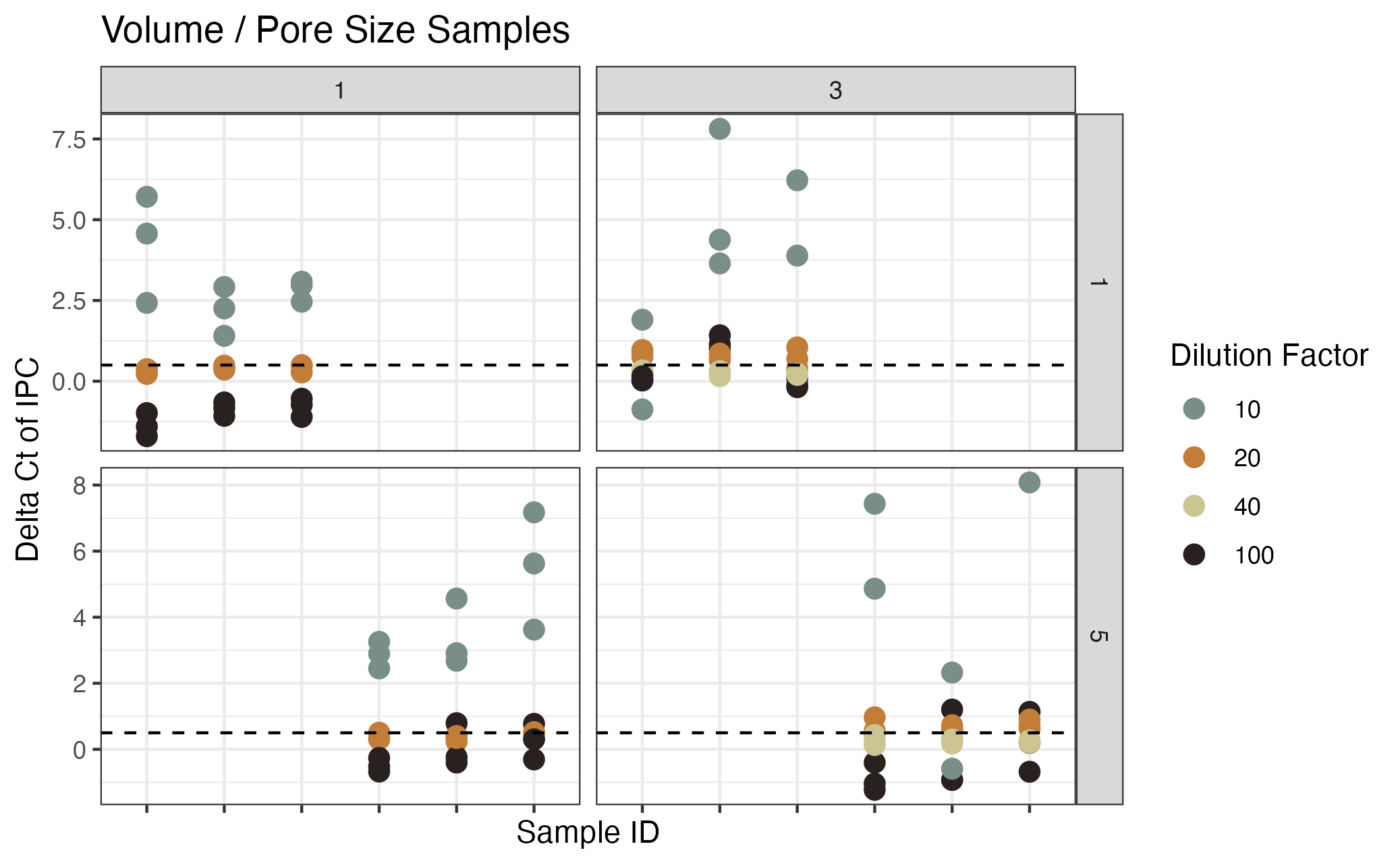

Supplement: Supplemental Information 2 — The y axis shows the difference in Ct value from the environmental sample versus the no template control of the spiked internal positive control (IPC). The x axis shows each unique sample. The facets correspond to the pore size of the filter (um) and shapres correspond to the volume of water filtered (L). The colors show the dilution factor. [file peerj-13-20127-s002.png]

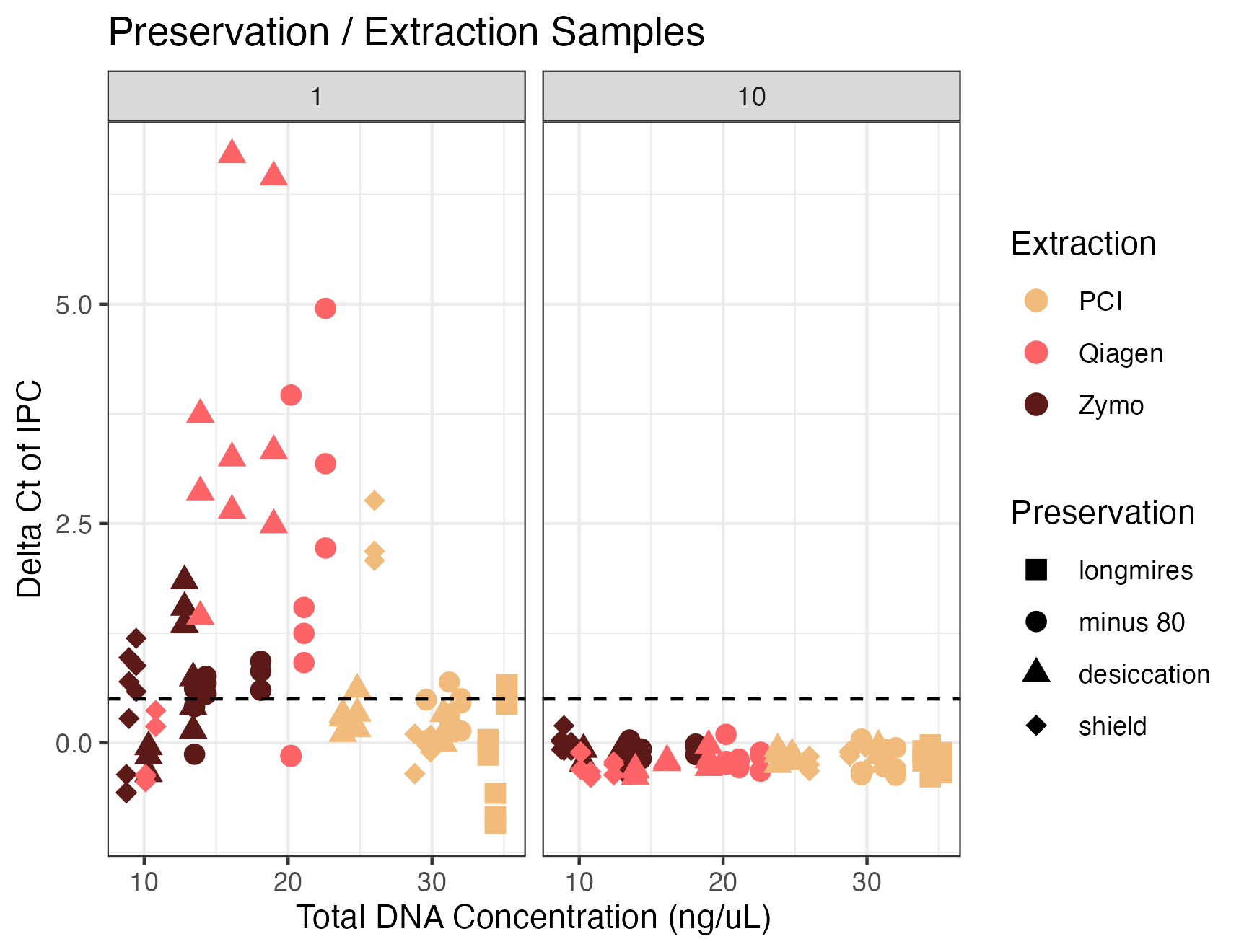

Supplement: Supplemental Information 3 — The y axis shows the difference in Ct value from the environmental sample versus the no template control of the spiked internal positive control (IPC). The x axis shows the total DNA concentration of the sample as measured by Qubit. Colors correspond to the extraction method and shapes correspond to the preservation method. Dashed lines represent the threshold at which samples were deemed inhibited (0.5 Ct difference). Facets indicate the dilution factor. [file peerj-13-20127-s003.png]

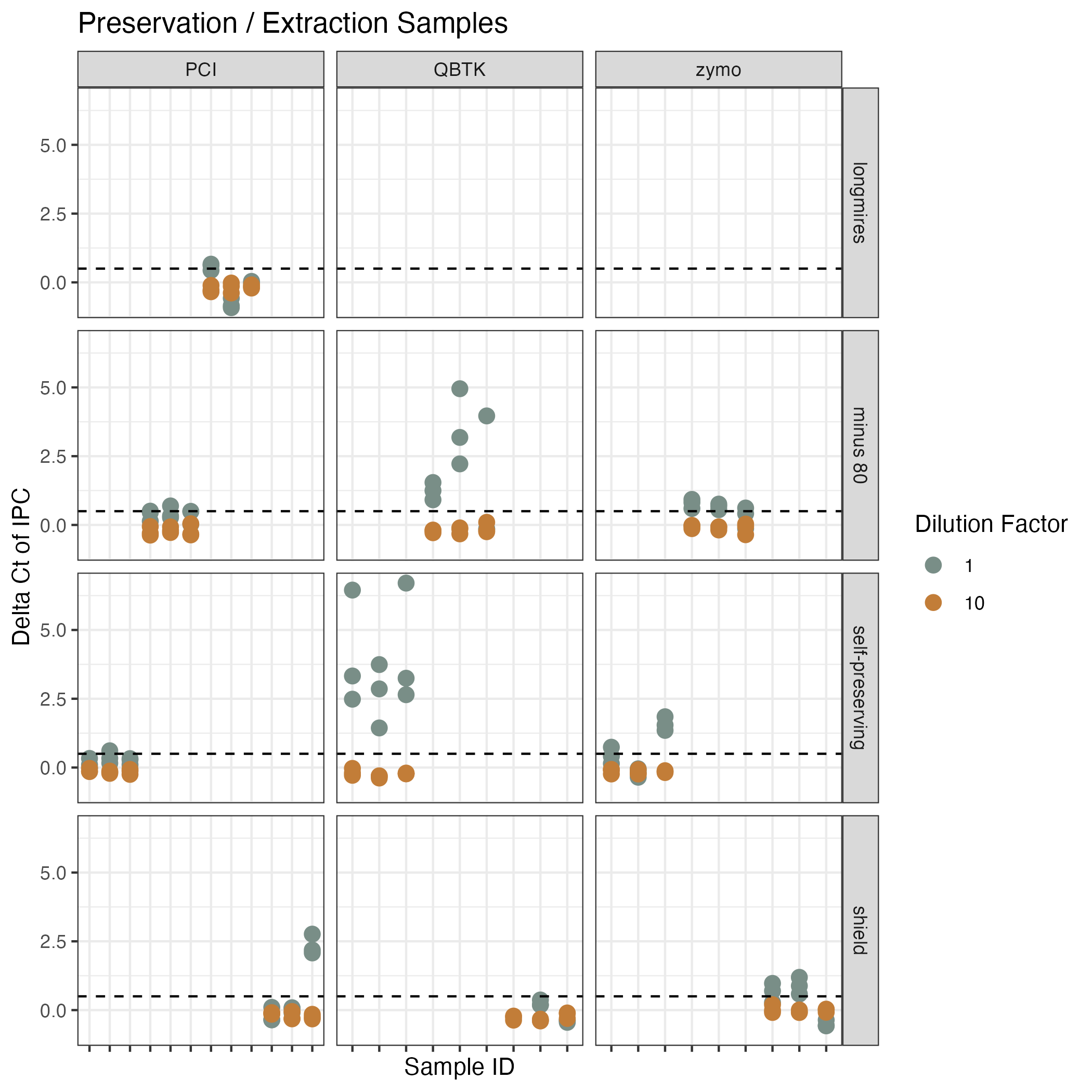

Supplement: Supplemental Information 4 — The y axis shows the difference in Ct value from the environmental sample versus the no template control of the spiked internal positive control (IPC). The x axis shows each unique sample. The facets correspond to the preservation and extraction method. The colors show the dilution factor. [file peerj-13-20127-s004.png]

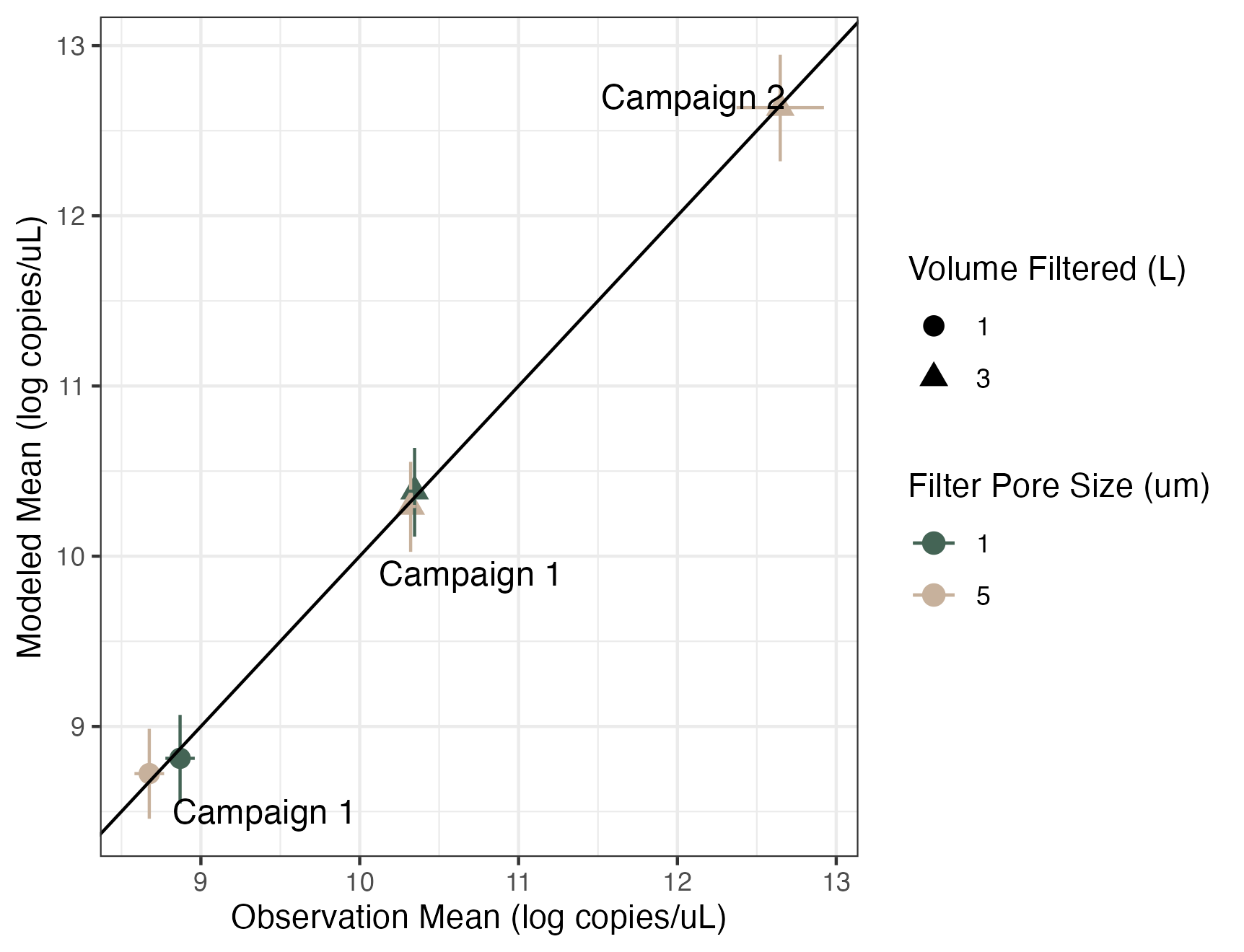

Supplement: Supplemental Information 6 — Modeled estimates versus the observed mean of technical and biological replicates. Error bars show 2.5% and 97.5% confidence intervals. Colors correspond to the pore size of the filter (um) and shapes correspond to the volume of water filtered (L). [file peerj-13-20127-s006.png]

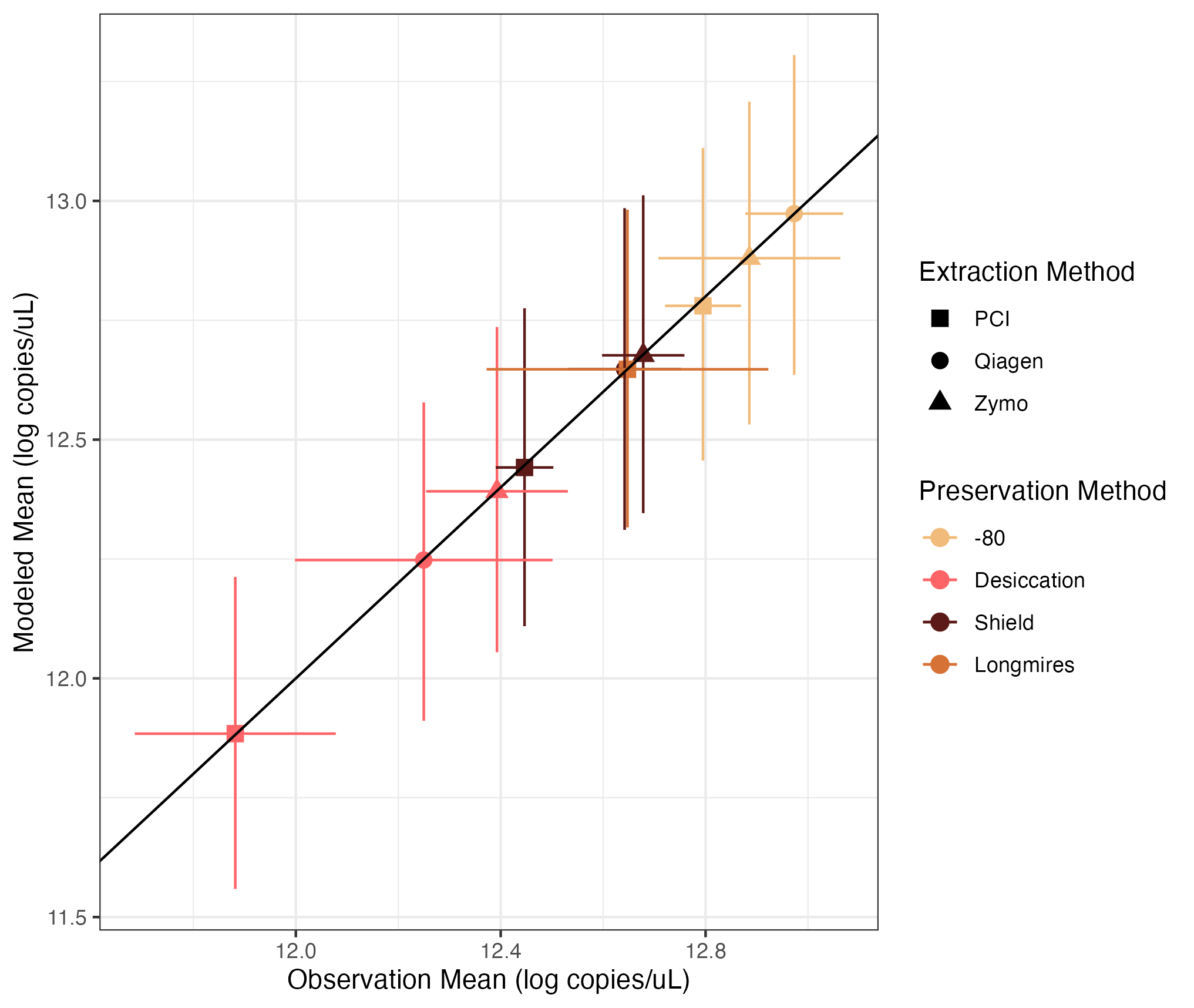

Supplement: Supplemental Information 7 — Modeled estimates versus the observed mean of technical and biological replicates. Error bars show 2.5% and 97.5% confidence intervals. Colors correspond to the extraction method and shapes correspond to the preservation method. [file peerj-13-20127-s007.png]
